# Neurodevelopmental Disorders (NDDs): Beyond the Clinical Definition and Translational Approach

**DOI:** 10.3390/children10010099

**Published:** 2023-01-03

**Authors:** Stefano Pallanti, Luana Salerno

**Affiliations:** 1 Istituto di Neuroscienze (INS), 50121 Florence, Italy; 2 Department of Psychiatry, Albert Einstein College of Medicine, Bronx, NY 10461, USA

Neurodevelopmental disorders (NDDs) are complex and heterogeneous disorders that affect the growth and development of the brain and are often associated with impairments in cognitive and motor functions, communication, and adaptive behavior. Although the diagnostic manuals define NDDs as separate entities, they co-occur with high frequency, determining a more complex clinical presentation, and therefore a more challenging evaluation and treatment. Moreover, they are frequently associated with other health conditions, causing increased negative individual and family burden and very high costs. Thus, the aim of this Special Issue of *Children* was to shed light on recent data in the field of NDDs, with regard to the etiopathogenesis of these disorders, as well as common and specific underpinnings, in order to raise awareness about the need for a deeper awareness of the specificities within these disorders and their common transdiagnostic mechanisms, with the goal of identifying new therapeutic targets and options. 

Pallanti and colleagues [1] investigated the use of transcranial photobiomodulation (tPBM) as a home-based nonpharmacological treatment for children and adolescents with ASD. Twenty-one children and adolescents aged 5–15 with a DSM-5 diagnosis of ASD were administered tPBM as an add-on to ongoing behavioral or pharmacological treatments. After 6 months of tPBM treatment, delivered at home 5 days per week, tPBM was associated with a reduction in ASD severity, as measured by CARS, in noncompliant behavior and parental stress, decreased behavioral and cognitive rigidity, and improvement in attentional functions and sleep quality. 

Marcotulli and colleagues [2] examined the relationship between the presence of EEG paroxysmal alterations and the metabolic profile of plasma amino and urinary organic acids in 55 children with ASD, aged 25–203 months. Authors found EEG epileptiform abnormalities associated with lower plasmatic levels of THR, and higher urinary levels of HMGA in children with ASD. Such findings provided support not only to the possible pathogenetic role of a THR deficiency in the brain excitatory/inhibitory imbalance characterizing ASD, but also to the hypothesis that abnormal brain activity may be associated with mitochondrial dysfunction. These findings may lead to new treatment aims.

Palomo-Carriòn et al. [3] have addressed the important issue of family involvement for the knowledge of the basic elements for the early intervention of children with unilateral cerebral palsy (UCP) (age < 12 months). Through semi-structured interviews, they have identified the barriers in early intervention, but also the possible facilitators of intervention programs. This work reiterated the importance of including families from the onset of the intervention planning to consider all the needs of the children and their families.

Salerno and colleagues [4] examined the state of the art related to the comorbidity between Attention Deficit Hyperactivity Disorder (ADHD) and Gaming Disorder (GD). Authors findings not only confirmed the frequent co-occurrence of ADHD and GD, but also that ADHD is associated with poor GD clinical outcomes, leading authors to state that gaming in children and adolescents with ADHD constitutes a pathological complication and not a functional coping strategy. They discussed findings within the Research Domain Criteria (R-Do-C) framework, describing the involvement of specific domains in the phenomenology of GD in ADHD, with the aim of highlighting the importance of clear identification of the brain circuits involved to design personalized interventions. 

Gaur [5] addressed the issue of comorbidity between ADHD and autoimmune disorders, particularly celiac disease (CeD). In her systematic review including 23 studies, 13 showed a positive association between ADHD and CeD, and most of them were published in the last 5 years. The author paid particular attention to the inattentive aspects of ADHD, and to the recent construct of Sluggish Cognitive Tempo (SCT), highlighting the importance of investigating the presentation (or subtype) of ADHD to improve knowledge about the phenotypic characteristics of the CeD when comorbid with ADHD. 

NDDs constitutes a public health challenge, and there is an urgent need to reduce misdiagnosis and providing new and appropriate treatment in the framework of personalized medicine. We believe this Special Issue contributes to provide useful insight to better face the NDDs complexity in both assessment and treatment, considering the influence of other psychiatric and somatic disorders that commonly lead to complex psychopathology and clinical presentation. 
